# CD47 antisense oligonucleotide treatment improves glucose homeostasis and alleviates dyslipidemia in aged male mice

**DOI:** 10.18632/aging.206343

**Published:** 2025-12-01

**Authors:** Taesik Gwag, Alana Newcomb, Josephine Otuagomah, Sue Murray, Shuling Guo, Sheng Tong, Philip A. Kern, Shuxia Wang

**Affiliations:** 1 Department of Pharmacology and Nutritional Sciences, University of Kentucky, Lexington, KY 40536, USA; 2 Lexington Veterans Affairs Medical Center, Lexington, KY 40502, USA; 3 Ionis Pharmaceuticals, 2255 Gazelle Court, Carlsbad, CA 92010, USA; 4 Department of Biomedical Engineering, University of Kentucky, Lexington, KY 40536, USA; 5 Internal Medicine, Endocrinology Division, University of Kentucky, Lexington, KY 40536, USA

**Keywords:** CD47 ASO, aging, metabolic disorder, hyperlipidemia, glucose homeostasis

## Abstract

As the global elderly population grows, age-associated metabolic disorders pose increasing public health challenges, highlighting the need for effective therapies. CD47, a transmembrane protein involved in immune and metabolic regulation, has been previously implicated in aging-related metabolic dysfunction. In this study, we investigated whether targeting CD47 by antisense oligonucleotide (ASO) could improve metabolic health in aged male mice. Twenty-month-old male mice were treated with control ASO or CD47 ASO (25 μg/g) for 10 weeks. We found that CD47ASO treatment selectively reduced visceral adiposity without affecting overall body weight in aged mice. It also improved glucose tolerance, insulin sensitivity, and hyperlipidemia—key metabolic disturbances commonly associated with aging. Mechanistically, CD47 ASO treatment reduced adipocyte size in visceral fat by suppressing lipogenesis rather than enhancing lipolysis, which was confirmed *in vitro* 3T3-L1 adipocyte model. It also stimulated hepatic glucose metabolism and improved brown adipose tissue (BAT) function by upregulating the expression of genes related to thermogenesis and endocrine signaling, including UCP1, CPT1β, and Neuregulin 4 (NRG4). Together, these findings support a beneficial role for CD47 knockdown in alleviating age-associated metabolic dysfunction through coordinated effects across multiple organs.

## INTRODUCTION

The global increase in the elderly population presents major challenges for public health systems [1–3]. Aging is a major risk factor for metabolic disorders, including obesity, type 2 diabetes, cardiovascular disease, and fatty liver disease [4–10]. Changes in body composition in aging, characterized by loss of muscle mass (sarcopenia) and increased fat mass, contribute to aging-associated metabolic syndrome [11–15]. Because physical activity is often limited in the elderly, comprehensive treatment strategies for obesity and metabolic disorders in aging populations, including new therapies, are needed [15].

CD47, a transmembrane protein, is upregulated in various cell types under aging conditions [16–19]. It is involved in cell proliferation, stress response, and inflammation [20]. CD47 is a well-established immune-regulatory protein that influences macrophage activation, phagocytosis, and inflammatory signaling via interactions with SIRPα or TSP1 [21–25]. Recently, anti-CD47 antibodies have been used in clinical trials to treat various cancers by targeting the “don’t eat me” signal, which involves the interaction between CD47 on cancer cells and SIRPα on macrophages [26–30]. Beyond cancer immunity, the role of CD47 has also been explored in other disease contexts [31]. Our lab and others have uncovered a novel role for CD47 signaling in the development of obesity and associated metabolic disorders, including chronic inflammation, insulin resistance, and fatty liver disease in adult mice [32–39]. In addition, CD47 signaling has been implicated in aging-related metabolic dysfunction [18, 35, 38]. Our previous studies demonstrated that global CD47 deficiency promoted white fat browning and activated brown fat thermogenesis, resulting in increased energy expenditure, reduced fat mass, and improved aging-related glucose intolerance and cold intolerance in aged male mice [35]. These metabolic benefits were confirmed in aged male mice but not female mice with brown adipocyte-specific CD47 deficiency [38]. Together, these findings suggest that targeting CD47 may represent a promising therapeutic strategy to improve aging-associated metabolic disorders. To test this hypothesis, CD47 ASO treatment was applied to naturally aged male mice.

In this study, we showed that CD47 ASO treatment in aged male mice selectively reduced visceral fat, improved glucose tolerance, insulin sensitivity, and lowered lipid levels. These benefits are linked to suppressed lipogenesis, reduced adipocyte size, increased anti-inflammatory macrophage markers in white fat, enhanced thermogenic and endocrine gene expression in brown fat, and upregulated glucose metabolism genes in the liver. Together, these results suggest that targeting CD47 may help combat aging-related metabolic dysfunction.

## RESULTS

### CD47ASO treatment reduced visceral fat mass without significantly affecting body weight in aged male mice

**Figure 1:**
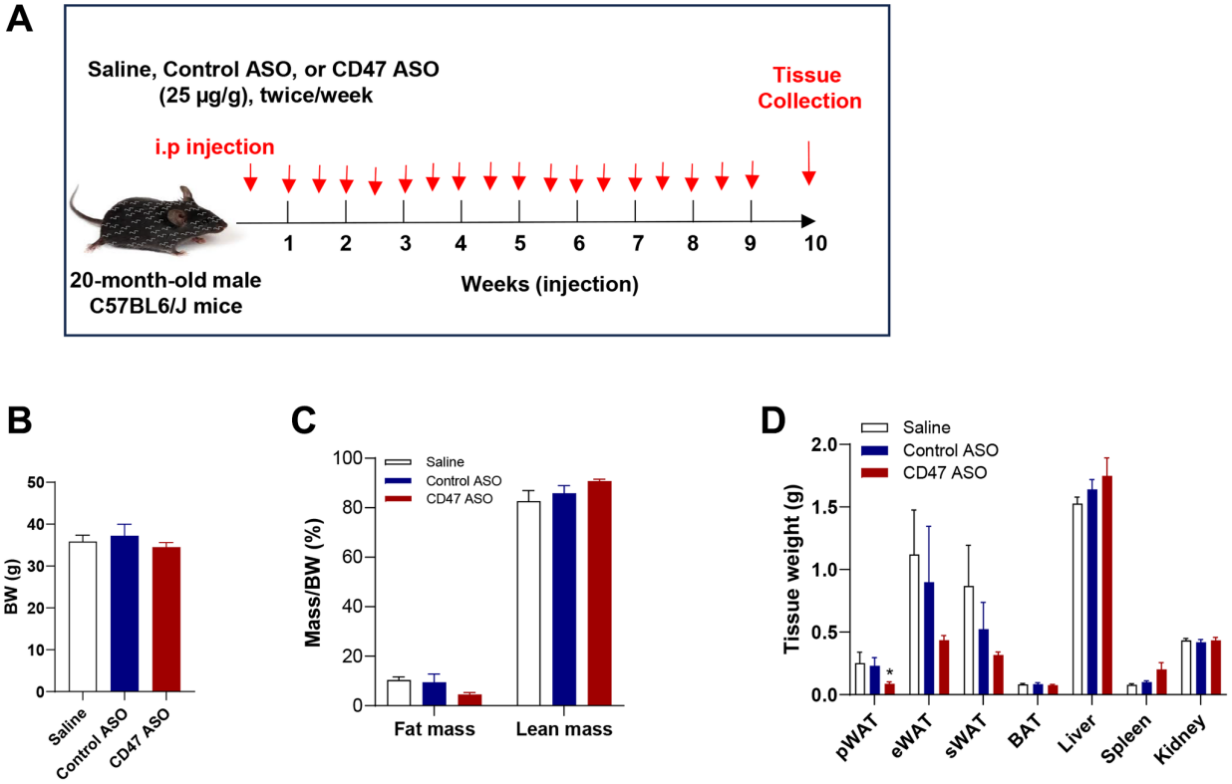
**CD47 ASO treatment reduced visceral fat mass in aged male mice.** (**A**) Experimental design. Twenty-month-old male C57BL/6J mice were fed a normal chow diet and treated with 25 μg/g of control ASO or CD47 ASO twice per week for 10 weeks; (**B**) Body weight; (**C**) Body composition was measured by EchoMRI; (**D**) Tissue weights were measured during collection. Data are represented as mean ± SEM (*n* = 6 mice/group). ^*^P < 0.05 compared to control ASO. Abbreviations: pWAT: peri-renal visceral white adipose tissue; eWAT: epididymal white adipose tissue; sWAT: subcutaneous white adipose tissue.

To evaluate the effect of CD47 ASO treatment on body weight and adiposity in aged mice, 20-month-old male C57BL6/J mice were administered saline, control ASO or CD47 ASO for 10 weeks (Figure 1A). At the end of the treatment period, body weight was measured and found to be comparable among three groups (Figure 1B). Body composition was measured by EchoMRI. There was a significant reduction in perirenal fat (pWAT, a visceral fat depot) in CD47 ASO group compared to control or saline group (Figure 1D). The total and epididymal fat (eWAT, another visceral fat depot) were also lower in CD47 ASO group but did not reach statistical significance (Figure 1C, 1D). Together, these data indicate that CD47 ASO treatment selectively reduces visceral adiposity without affecting overall body weight in aged male mice.

### CD47 ASO treatment improved glucose homeostasis and reduced hyperlipidemia in aged male mice

**Figure 2:**
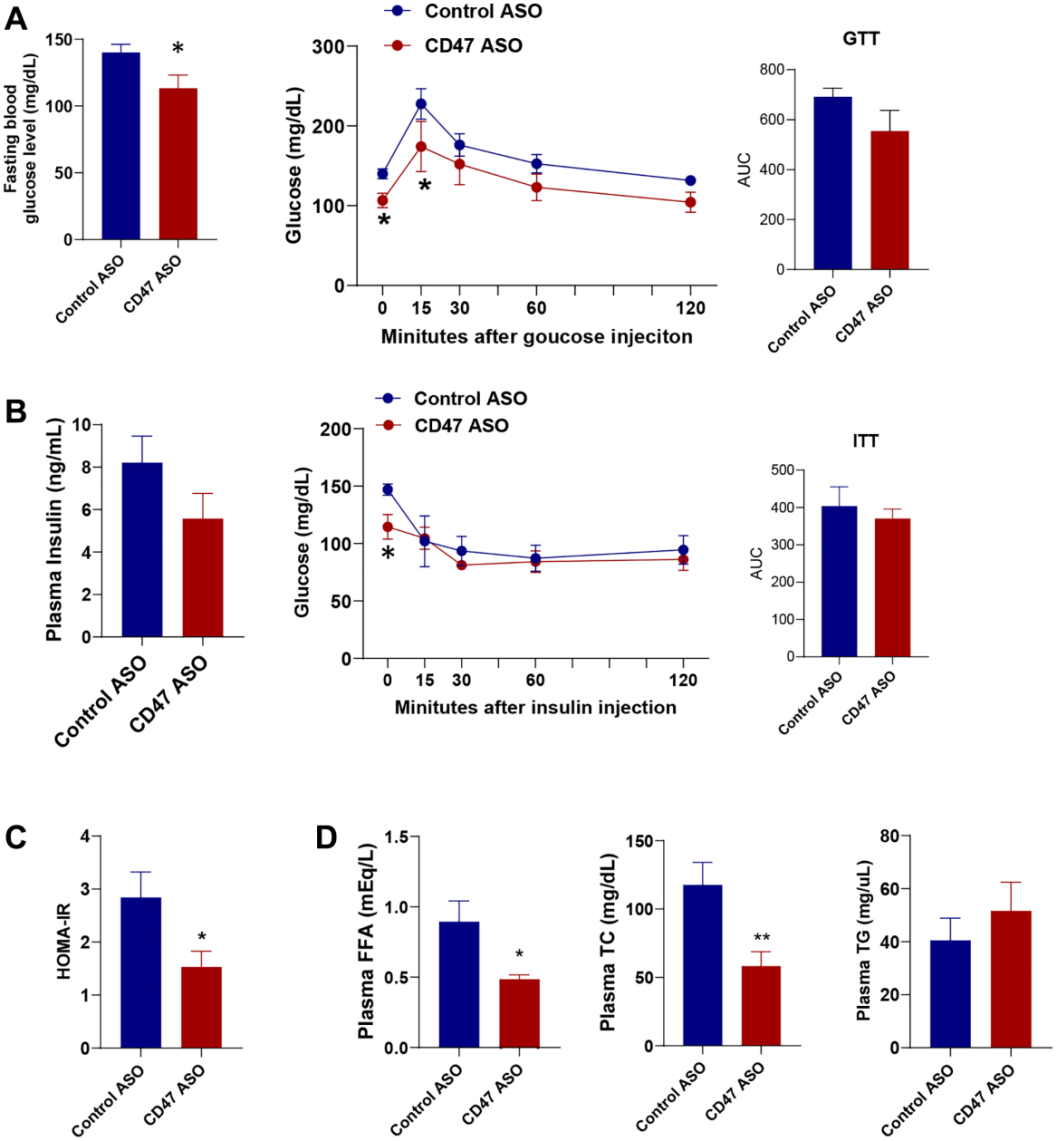
**CD47 ASO treatment improved glucose homeostasis and hyperlipidemia in aged male mice.** (**A**) Fasting glucose levels were measured after 6 hrs fasting and glucose tolerance test (GTT) was performed. AUC (area under curve of GTT) was shown; (**B**) Fasting insulin levels, Insulin tolerance test (ITT) and AUC of ITT were shown; (**C**) HOMA-IR was calculated; (**D**) Plasma free fatty acid, total cholesterol, and triglycerides levels were measured. Data are represented as mean ± SEM (*n* = 6) mice/group). ^*^P < 0.05 and ^**^P < 0.01 compared to control ASO group.

Since we did not observe any differences between the saline and control ASO groups in body weight or fat mass (Figure 1), the following data presentation focuses specifically on the CD47 ASO and control ASO groups. To determine whether CD47 ASO treatment affects glucose homeostasis, we performed glucose tolerance and insulin tolerance tests. We found that fasting blood glucose levels were significantly lower in CD47 ASO-treated mice compared to controls, accompanied by improved glucose tolerance (Figure 2A). Although plasma insulin levels and insulin tolerance were not markedly reduced in the CD47 ASO group (Figure 2B), the HOMA-IR (Homeostatic Model Assessment for Insulin Resistance) index was significantly decreased following CD47 ASO treatment (Figure 2C), indicating enhanced insulin sensitivity. Aging is often associated with hyperlipidemia such as high cholesterol levels [40–42]. Interestingly, CD47 ASO treatment led to a reduction in hyperlipidemia: plasma levels of free fatty acids (FFA) and total cholesterol were significantly decreased in the CD47 ASO group (Figure 2D). These data indicate that CD47 ASO treatment improves aging-related insulin resistance and hyperlipidemia.

### CD47 ASO treated aged male mice had reduced adipocyte size in visceral white adipose tissue

**Figure 3:**
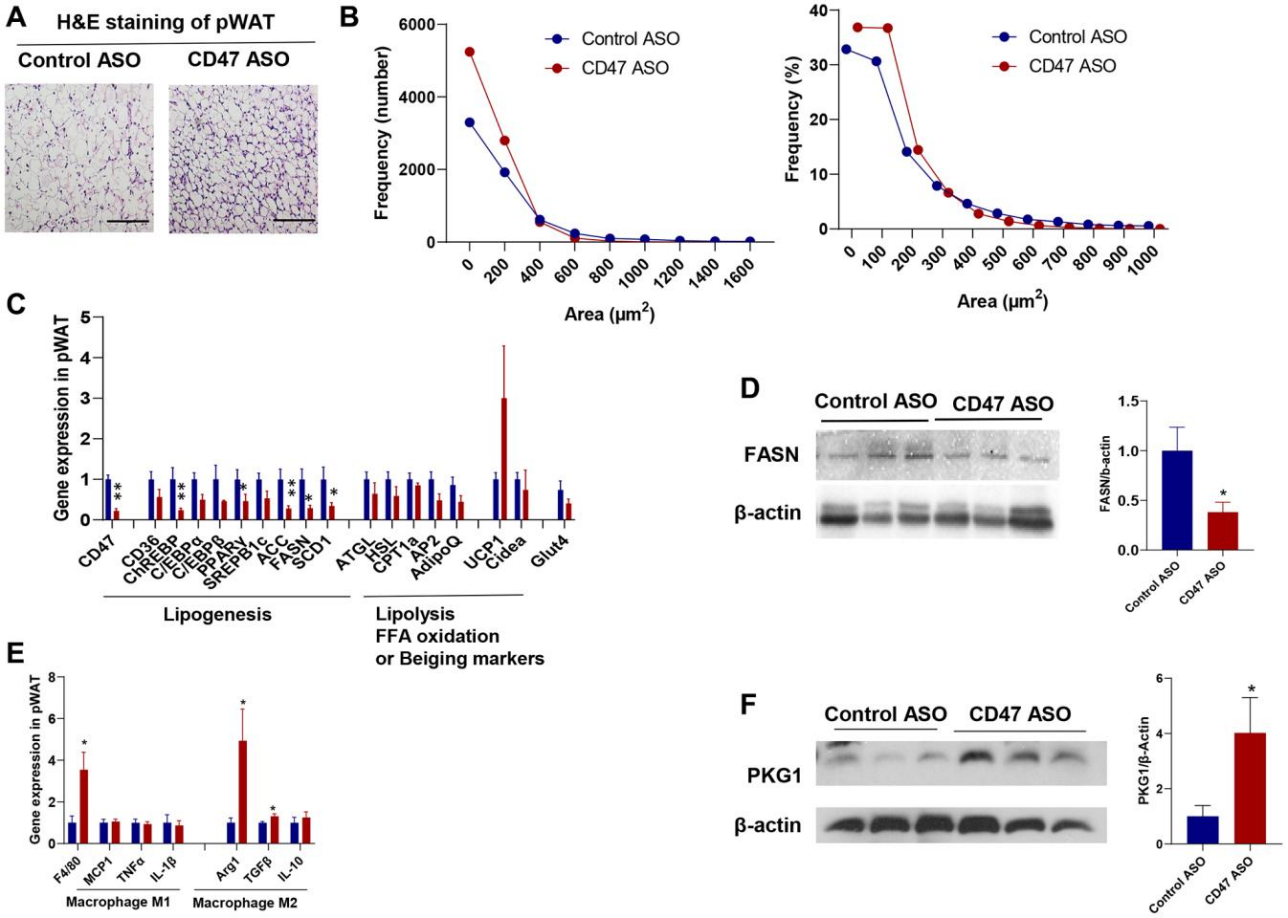
**CD47 ASO treatment reduced adipocytes size of visceral fat by suppressing lipogenesis.** (**A**) Representative H&E staining image of peri-renal white adipose tissue (pWAT) (scale bar = 100 μm); (**B**) Frequency of adipocytes size of pWAT; (**C**) Gene expression in pWAT by qPCR; (**D**) FASN protein expression level by immunoblotting and quantification; (**E**) Inflammatory and macrophage markers gene expression in pWAT by qPCR. (**F**). PKG1 protein expression level by immunoblotting and quantification. Data are represented as mean ± SEM (*n* = 6 mice/group). ^*^P < 0.05 and ^**^P < 0.01 compared to control ASO group.

Visceral fat increases with age and is a major risk factor for insulin resistance, type 2 diabetes, and cardiovascular mortality [43, 44]. We found that pWAT mass was significantly reduced in CD47 ASO treated aged mice compared to controls (Figure 1D). To further characterize pWAT, we performed hematoxylin and eosin (H&E) staining, quantified adipocyte size, and analyzed the frequency distribution. As shown in Figure 3A, 3B, adipocytes in pWAT from CD47 ASO-treated mice were smaller than those from the control ASO group. CD47 knockdown in pWAT following CD47 ASO treatment was confirmed by qPCR (Figure 3C).

To investigate the mechanisms of reduced adipocytes size in CD47ASO treated mice, qPCR was performed to determine the expression of genes relating to lipid metabolism in pWAT. As shown in Figure 3C, lipolysis related genes or fatty acid oxidation genes were comparable between two groups; while the lipogenesis-related genes such as ChREBP (carbohydrate response element-binding protein), Acetyl-CoA Carboxylase (ACC), Fatty acid synthase (FASN, the key rate-limiting enzyme in *de novo* lipogenesis), and Stearoyl-CoA desaturase 1 (SCD1) were significantly downregulated in CD47 ASO group compared to control ASO group. Consistently, FASN protein levels were also reduced in pWAT from CD47 ASO-treated mice (Figure 3D). A similar reduction in lipogenesis gene expression was also observed in epididymal fat from CD47 ASO-treated mice (data not shown). We also examined beiging markers (e.g., UCP1, Cidea) and found that CD47 ASO treatment induced a trend toward increased UCP1 expression in pWAT (Figure 3C). Together, these findings suggest that the reduced adipocyte size in visceral fat following CD47 ASO treatment might be not due to increased lipolysis, but rather to suppressed lipogenesis. CD47 is known to influence several signaling pathways, including the cAMP/PKA and cGMP/PKG pathways [45–48]. To identify which pathways are involved in pWAT, we measured cAMP levels and found no significant differences between CD47 ASO and control ASO treated mice (data not shown). The cGMP/PKG pathway has been reported to promote adipocyte differentiation and thermogenesis, thereby regulating metabolism [49–53]. Moreover, our previous studies showed that cGMP/PKG signaling was upregulated in metabolic tissues from CD47−/− mice [34]. Therefore, we examined PKG1 protein levels in pWAT. Consistently, increased PKG1 was shown in pWAT from CD47 ASO treated mice (Figure 3F). This finding aligns with the healthier adipose tissue phenotype observed in CD47 ASO treated mice.

Additionally, we found that CD47 ASO treatment may promote a shift in adipose tissue macrophage phenotype toward an anti-inflammatory M2 state. Gene expression of the macrophage marker F4/80 was increased in pWAT from CD47 ASO-treated mice. Notably, we observed upregulation of M2 macrophage markers such as Arg1 and TGF-β, while proinflammatory M1 markers were comparable between two groups (Figure 3E).

### CD47 ASO treatment reduced lipogenesis in mature 3T3-L1 cell–derived white adipocytes differentiated for 15 days *in vitro*

**Figure 4:**
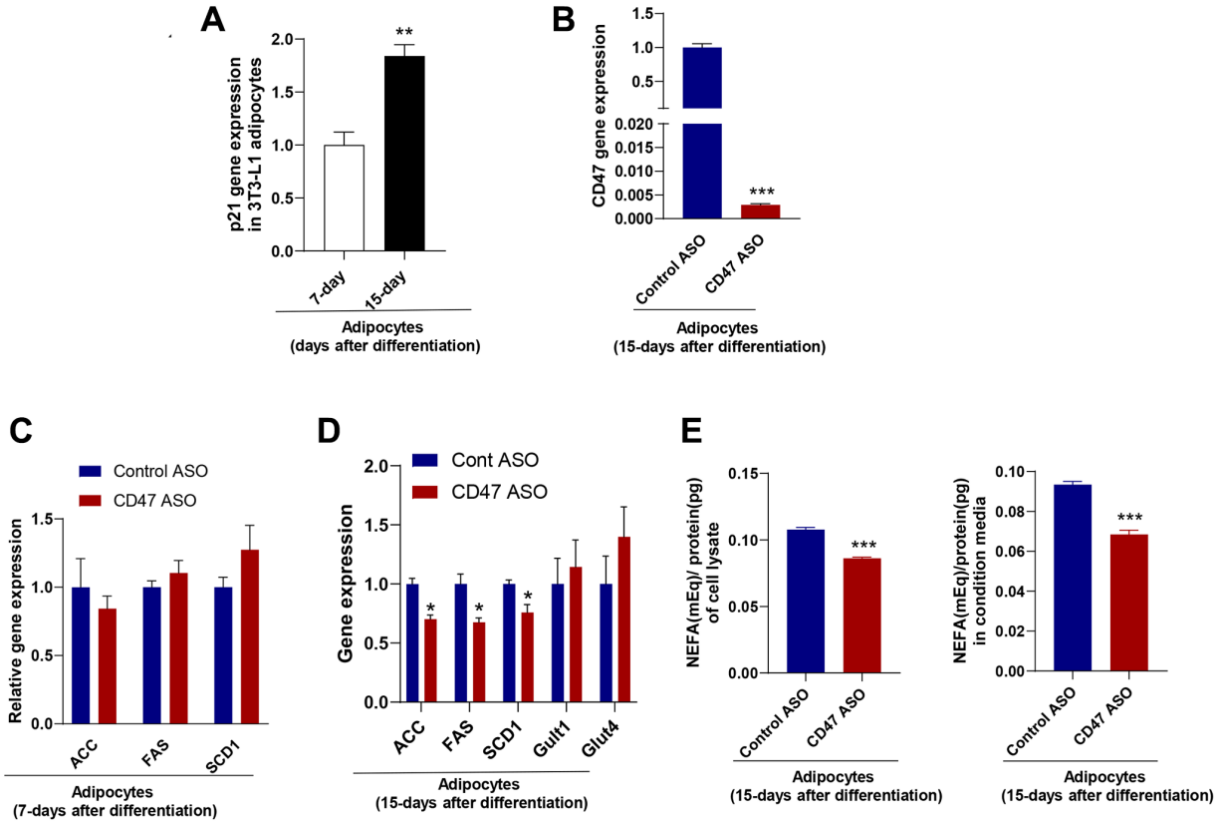
**CD47 ASO treatment reduced de novo lipogenesis in 3T3-L1 adipocytes (after 15 days of differentiation).** (**A**) Expression of the senescence marker, p21 in 15 days differentiated 3T3-L1 adipocytes (modeling adipocytes from old mice) compared to 5–7 days differentiated cells (modeling adipocytes from young mice); (**B**–**D**) CD47 or other gene expression in 7-day or 15-day differentiated adipocytes after 48 hours of treatment with the control ASO or CD47ASO (100 μM); (**E**) De novo lipogenesis measured in cell lysate and conditional medium from 15-day differentiated adipocytes. Data are represented as mean ± SEM (n = 3/group). ^**^P < 0.01 compared to young group; ^*^P < 0.05 and ^***^P < 0.001 compared to control ASO group.

To further confirm the inhibitory effect of CD47 knockdown on lipogenesis in mature white adipocyte *in vitro*, we utilized a 3T3-L1 cell line. As previously described [54], 3T3-L1 cells were differentiated into mature white adipocytes and cultured for more than 15 days to induce senescence, serving as an *in vitro* model to mimic adipocytes isolated from aged mice. Senescence was confirmed by increased p21 gene expression compared to 7-day–differentiated adipocytes (mimic adipocytes from young mice) (Figure 4A). Using this white adipocyte model (15 days after differentiation), we examined the effect of CD47 knockdown on lipogenesis. CD47 ASO treatment significantly reduced CD47 gene expression in these adipocytes (Figure 4B). The expression of lipogenesis-related genes, including ACC, FASN, and SCD1, was not affected in adipocytes after 7 days of differentiation (Figure 4C), but was significantly downregulated in adipocytes after 15 days of differentiation following CD47 ASO treatment (Figure 4D). Furthermore, glucose-stimulated lipogenesis was assessed, and as shown in Figure 4E, the levels of *de novo*–synthesized free fatty acids (FFAs) were reduced in both the cell lysate and the conditioned medium of CD47 ASO–treated adipocytes after 15 days of differentiation.

We also examined whether CD47 ASO treatment affects 3T3-L1 adipogenesis. 3T3-L1 cells were treated with either Control ASO (100 μM) or CD47 ASO (100 μM) and induced differentiation. Five days after differentiation, Oil Red O staining and qPCR analyses were performed. As shown in Supplementary Figure 1, Oil Red O staining revealed a trend toward increased lipid accumulation in CD47 ASO treated cells. Consistently, the expression of adipogenic markers, including aP2, PPARγ, and perilipin, as well as lipogenic genes such as ACC, FAS, and SCD1, was elevated, suggesting that CD47 ASO treatment promotes 3T3-L1 adipogenesis *in vitro*. Collectively, these data suggest that CD47 ASO treatment promotes preadipocyte differentiation while exerting maturation-dependent effects on lipogenic gene expression in adipocytes, enhancing lipid accumulation in early differentiation but suppressing lipogenesis in senescent adipocytes.

### CD47 ASO treatment upregulated the expression of thermogenic and batokine genes in BAT of aged male mice

**Figure 5:**
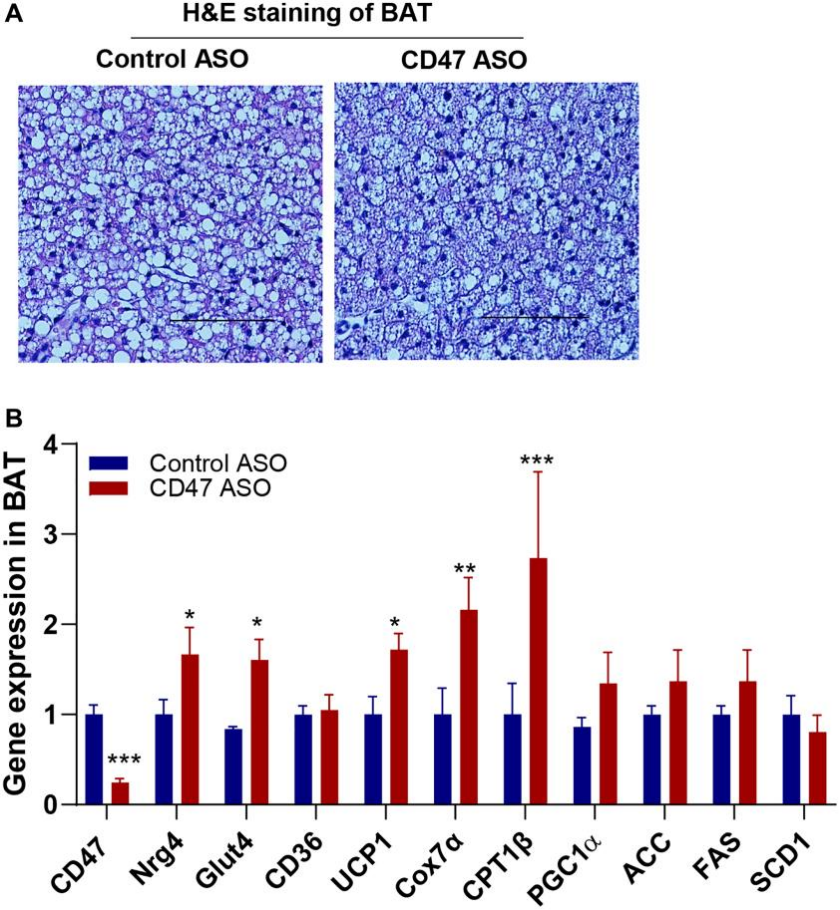
**CD47 ASO treatment stimulated the expression of mitochondrial and thermogenesis-related genes in BAT from aged male mice.** (**A**) Representative H&E staining image of BAT (scale bar = 100 μm); (**B**) Gene expressions in BAT by qPCR. Data are represented as mean ± SEM (n=6 mice/group). ^*^P < 0.05, ^**^P < 0.01 and ^***^P < 0.001 compared to control ASO group.

In our previous studies, we found that global CD47 deficiency enhanced brown fat thermogenic function in both young and aged male mice, which was associated with improved metabolic health [34, 35, 38]. In the present study, we investigated whether CD47 ASO treatment would produce similar benefits in brown fat. We found that CD47 ASO treatment did not affect BAT mass (Figure 1D) or morphology (Figure 5A). Expression of lipogenic genes (ACC, FAS, or SCD1) in BAT was unaffected by CD47ASO treatment (Figure 5B). However, it significantly increased the expression of genes related to thermogenesis, fatty acid beta oxidation and mitochondrial function, including UCP1, CPT1beta, and Cox7α (cytochrome c oxidase subunit 7 alpha) (Figure 5B). Interestingly, Glut4 and Neuregulin 4 (NRG4), a batokine, were also upregulated in CD47 ASO–treated mice. (Figure 5B). NRG4 is secreted by BAT and acts on other organs, such as the liver, to regulate lipid and glucose metabolism [55, 56]. Collectively, these findings suggest that CD47 ASO treatment enhances the thermogenic and possibly endocrine functions of brown fat, contributing to improved systemic metabolic health, including better glucose homeostasis in aged mice.

### CD47 ASO treatment enhanced the expression of genes involved in hepatic glucose metabolism without affecting hepatic lipid accumulation in aged male mice

**Figure 6:**
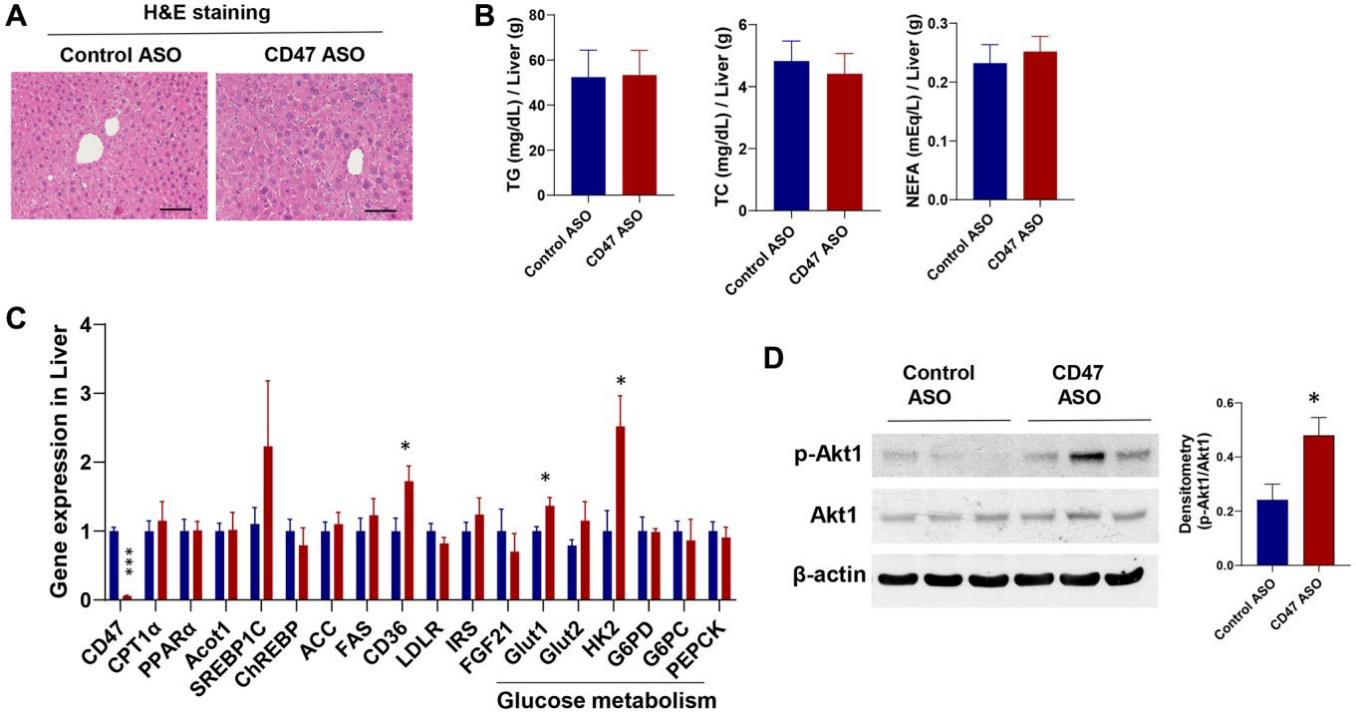
**Effect of CD47 ASO treatment on liver histology and gene expression in aged male mice.** (**A**) Representative H&E staining image of liver (scale bar = 100 μm); (**B**) Hepatic lipid levels including triglycerides, total cholesterol, and free fatty acid; (**C**) Gene expression in liver by qPCR; (**D**) Representative hepatic immunoblotting of p-AKT1, total AKT, and β-actin, with corresponding densitometry results. Data are represented as mean ± SEM (*n* = 6 mice/group). ^*^P < 0.05 and ^***^P < 0.001 compared to control group.

To assess the impact of CD47 ASO treatment on liver phenotype in male aged mice, we first examined histology and lipid content. Hematoxylin and eosin (H&E) staining showed no obvious morphological changes between CD47 ASO and control ASO treated mice (Figure 6A). Consistently, measurements of hepatic triglycerides (TG), total cholesterol (TC), and non-esterified fatty acids (NEFA) revealed no significant differences between the two groups (Figure 6B). Gene expression analysis by qPCR showed that lipogenic genes were unchanged, whereas glucose metabolism–related genes, including Glut1 (glucose transporter 1) and HK2 (Hexokinase 2), were significantly upregulated in CD47 ASO treated mice (Figure 6C). Glut2 showed a trend toward increased expression. Moreover, phosphorylation of AKT1 was increased in the liver following CD47 ASO treatment (Figure 6D). Together, these data suggest that enhanced hepatic glucose metabolism may contribute to the improved systemic glucose tolerance observed in these CD47 ASO treated mice.

## DISCUSSION

In this study, we investigated the therapeutic potential of CD47 ASO treatment in aging-related metabolic dysfunction in naturally aged male mice. We found that CD47 ASO treatment improved overall metabolic health in aged male mice, marked by reduced visceral fat mass, improved glucose homeostasis, and reduced hyperlipidemia. These metabolic benefits are associated with suppressed lipogenesis in visceral white fat, increased thermogenic and endocrine activity in brown fat, and enhanced hepatic glucose metabolism, suggesting that CD47 ASO may represent a novel therapeutic approach to counteract aging-related metabolic dysfunction.

One interesting finding of this study is that CD47 ASO treatment did not induce overall weight loss in aged mice, but it significantly reduced the mass of peri-renal visceral white adipose tissue (pWAT). This tissue contained a greater number of smaller adipocytes (Figure 3), which may be partly due to enhanced adipogenesis resulting from CD47 knockdown in preadipocytes (Supplementary Figure 1). The reduction in adipocyte size could also result from suppressed *de novo* lipogenesis (DNL) rather than increased lipolysis, as CD47 ASO treatment downregulated key DNL genes such as ACC and FASN, while expression of lipolysis-related genes remained largely unchanged (Figure 3). These observations were further validated in an *in vitro* model of 3T3-L1 adipocytes differentiated for 15 days to induce senescence (Figure 4), where CD47 ASO selectively reduced DNL in senescent adipocytes while sparing early differentiated cells (7-day differentiation). One potential mechanism is that CD47 inhibition may modulate senescence-associated pathways, including p53/p21 and NF-κB signaling, which are highly active in aged adipocytes [57–59]. In senescent cells, these pathways contribute to altered transcriptional programs and chronic low-grade inflammation that can interfere with lipid metabolism. By dampening p53/p21–mediated transcriptional repression and NF-κB–driven inflammatory signaling, CD47 ASO may downregulate lipogenic genes specifically in senescent adipocytes.

In addition to the non-canonical (cell-autonomous) effects of CD47 on adipocyte function (as described above), CD47’s canonical role as a ligand for macrophage SIRPα may also influence adipocyte function by mediating immune cell–adipocyte crosstalk *in vivo*. Consistent with this, CD47 ASO treatment reduced lipogenic gene expression in white adipose tissue of aged mice (Figure 3C), accompanied by increased macrophage infiltration and selective elevation of M2 markers. This shift toward a reparative macrophage phenotype may promote adipose tissue remodeling, enhance lipid turnover, and support healthier tissue architecture and metabolic flexibility [60, 61], which together likely contribute to improved insulin sensitivity and reduced adipocyte size. Collectively, these findings indicate that CD47 inhibition exerts complementary effects on adipocytes through both intrinsic and extrinsic pathways. Future studies employing flow cytometry, transcriptomic profiling of adipose-resident immune cells, and immune cell–adipocyte co-culture experiments will help clarify how CD47-targeted therapy reprograms immune function and senescence-associated signaling to support systemic metabolic health during aging.

DNL is a complex metabolic process that synthesizes fatty acids from non-lipid precursors, subsequently converting them into triglycerides [62, 63]. While DNL plays an essential role in maintaining cellular and whole-body energy homeostasis, its chronic elevation is associated with numerous pathological conditions, including type 2 diabetes, non-alcoholic fatty liver disease (NAFLD), cardiovascular disease, neurodegeneration, and aging [64–67]. As such, inhibitors targeting key DNL enzymes such as FASN and ACC have been explored as therapeutic strategies for various metabolic disorders [64, 68]. Under conditions of obesity or aging, DNL becomes dysregulated and can lead to adverse metabolic consequences, including insulin resistance, type 2 diabetes, and cardiovascular disease [63, 69–71]. Notably, impaired lipogenesis in WAT has been linked to reduced production of insulin-sensitizing fatty acids, contributing to systemic insulin resistance [72]. Paradoxically, genetic inhibition of adipose tissue lipogenesis, such as in adipose-specific FASN knockout mice, has been shown to promote white fat beiging, enhance energy expenditure, and protect against diet-induced obesity and insulin resistance [73]. Interestingly, CD47 ASO treatment in aged mice suppressed DNL in pWAT and was accompanied by a trend toward increased UCP1 expression at the current dosage (25 μg/g). It is possible that higher doses or longer treatment duration may be required to achieve a more pronounced beiging effect.

In contrast to its effects in adipose tissue, CD47 ASO treatment did not significantly alter the expression of key DNL genes in the liver (Figure 6) or brown fat (Figure 5), suggesting that hepatic lipogenesis or brown fat lipogenesis was unaffected. The mechanisms underlying this tissue-specific effects of CD47 ASO on DNL regulation under aging conditions warrant further investigation. Notably, the expression of ChREBP, a major transcriptional regulator of DNL, was reduced in white adipose tissue (Figure 3) but remained unchanged in the liver (Figure 6), suggesting that differential transcriptional control may contribute to the adipose-specific response. CD47 is known to influence several signaling pathways [45–48], which could regulate ChREBP activity in a tissue-dependent manner. Indeed, we observed increased PKG1 signaling in white fat (Figure 3F) but elevated AKT signaling in the liver (Figure 6D), highlighting distinct tissue-specific downstream effects of CD47 inhibition. Together, these findings indicate that white adipocytes may be particularly responsive to CD47 blockade due to their unique signaling environment and potentially age-related changes in transcriptional sensitivity. Future studies employing tissue-specific knockouts or ChREBP reporter systems will be important for delineating the molecular interactions between CD47 signaling and lipogenic transcription factors in aging adipose tissue.

We also determined the effect of CD47 ASO treatment on BAT function. Our previous studies demonstrated that global CD47 deficiency activated brown fat thermogenesis, resulting in increased energy expenditure, reduced fat mass, and improved aging-related glucose intolerance and cold intolerance in aged male mice [35]. These metabolic benefits were confirmed in aged male mice with brown adipocyte-specific CD47 deficiency [38]. Consistently, in this study, we found that CD47 ASO treatment downregulated CD47 expression in BAT and upregulated expression of mitochondria and thermogenic related genes such as UCP1, CPT1β (the rate-limiting enzyme in mitochondrial fatty acid β-oxidation) and Cox7α (Figure 5). This result suggests that CD47 ASO treatment may stimulate BAT fatty acid oxidation and increase thermogenesis. In addition, we also found that CD47ASO treatment upregulated Glut4 and NRG4 expression in BAT. NRG4, acting as a batokine, has been shown to improve metabolic diseases including type 2 diabetes, fatty liver disease, and atherosclerosis through its effect on multiple organs [56, 74–78]. This finding suggests that CD47ASO treatment may regulate BAT endocrine function and through interorgan crosstalk aging associated metabolic disorder is improved.

Interestingly, in this study, skeletal muscle function or phenotype in aged mice appeared largely unaffected by CD47 ASO treatment (Figure 1C and Supplementary Figure 2). Lean mass was not altered, and we did not observe significant changes in the expression of genes associated with cellular senescence (e.g., p21, p53), oxidative stress response (Nrf2), glucose transport (Glut1, Glut2), or lipid metabolism (PPARα) in skeletal muscle, suggesting a limited direct impact of CD47 knockdown on muscle tissue at the transcriptional level. This finding contrasts with a recent report indicating that blockade of TSP1–CD47 signaling enhanced muscle stem cell function and promoted muscle regeneration and strength in aged mice [16]. The discrepancy may be due to differences in experimental design or data analysis, such as the method or duration of CD47 inhibition (e.g., CD47 ASO versus TSP1 antibody), the presence or absence of acute injury or regenerative stimuli, or the use of whole-tissue analysis versus isolation of specific muscle stem cells for targeted evaluation. Further mechanistic studies are warranted to determine whether longer treatment duration, additional stress stimuli, or combination with exercise might unmask potential benefits of CD47 inhibition in skeletal muscle.

One limitation of the current study is that we focused on aged male mice; it is important to consider potential sex-specific effects of CD47 ASO treatment. Previous work from our group has shown that brown adipocyte–specific CD47 knockout protects male mice, but not female mice, from aging-related metabolic dysfunction [38]. These findings suggest that the metabolic benefits of CD47 inhibition may be male-specific, likely due to sex differences in brown adipose tissue activity and systemic energy metabolism. Such sex dimorphism may arise from multiple factors, including differential CD47 expression levels between males and females, variations in adipose immune cell composition and polarization states, and the influence of sex hormones or sex chromosome complement on adipose tissue remodeling and immune–metabolic communication. In future studies, it will be important to determine whether CD47 ASO treatment in aged female mice, or alternative therapeutic strategies, can confer similar protection and to elucidate the molecular mechanisms underlying these sex-dependent responses.

In summary, our findings demonstrate that CD47 ASO treatment exerts metabolically protective effects in aged male mice by reducing visceral adiposity, improving glucose and lipid homeostasis, and enhancing insulin sensitivity. These benefits occur without changes in total body weight and are associated with reduced lipogenesis in white adipose tissue, enhanced thermogenic and endocrine activity in brown fat, increased hepatic glucose metabolism, and modulation of the immune system. Together, these results highlight CD47 as a promising therapeutic target for age-related metabolic dysfunction.

## MATERIALS AND METHODS

### Mice and CD47 ASO treatment

Based on our previous studies showing that CD47 deficiency protected only male mice from aging-associated metabolic dysfunction [35, 38], in this study we tested the therapeutic effect of CD47 antisense oligonucleotide (ASO) in old male mice. All mice were housed in standard cages at 22°C under a 14:10-h light-dark cycle. Twenty-month-old C57BL/6 male mice under normal chow diet condition were injected twice a week with saline, control ASO (25 μg/g), or CD47 ASO (25 μg/g) (provided by Ionis Pharmaceuticals, Carlsbad, CA, USA) for 10 weeks (Figure 1A) as described in our previous study [33]. The body weight was monitored weekly. At the end of study, body composition (e.g., fat mass and lean mass) was measured by EchoMRI. Then mice were sacrificed, and plasma and tissues were collected for further process.

### Blood parameter analysis

The plasma insulin levels were measured using an ELISA kit (Crystal Chem, Elk Grove Village, IL, USA). The fasting blood glucose levels were measured using glucose monitoring system (US Diagnostics, Inc., Plantation, FL, USA) after 6 hr fasting during performance of insulin tolerance (ITT) or glucose tolerance test (GTT) as previously described [34]. In addition, the HOMA-IR was calculated using formula: fasting glucose levels (mg/dL) x fasting insulin levels (ng/mL)/405, as described previously [56].

### Lipid analysis

Total lipids from frozen liver were extracted as previously described [79]. Liver and plasma triacylglycerol (TG) and total cholesterol (TC) levels were measured enzymatically by using kits from the Wako Chemicals (Richmond, VA, USA). The FFA levels of plasma, 3T3-L1 cell lysate, and conditional medium were measured enzymatically by using kits from the Wako Chemicals (Richmond, VA, USA).

### Histology analysis

Tissues (Liver, WAT, BAT, and muscle) were fixed in 10% formalin overnight immediately after collection, then stored in 70% ethanol before paraffin embedding. H&E staining from fat or liver tissue was provided by Pathology Core at the University of Kentucky.

### Quantification of adipocytes size and frequency distribution

The analysis of the adipocytes size was performed as previously described [56]. The visceral adipose tissue H&E images were taken from 3 different random fields in each mouse (*n* = 6 per group). Over 200 adipocytes were counted in each field. Total 18 images were analyzed using Adiposoft in imageJ Software.

### *In vitro* 3T3-L1 adipocytes model

3T3-L1 preadipocyte from ATCC were maintained in DMEM medium including 10% FBS, 25 mM HEPES, 4.5 g/L glucose, and 100 U/ml penicillin/streptomycin at 37ºC in a 5% CO_2_. After 2 days from 100% confluence, 3T3-L1 cells were differentiated into adipocyte in DMEM including 10% FBS, 25 mM HEPES, 4.5 g/L glucose, 100 U/ml penicillin/streptomycin, 0.5 mM IBMX, 1.7 μM insulin, and 1 μM dexamethasone at 37ºC in a 5% CO_2_. We differentiated 3T3-L1 cells for 5–7 days to model adipocytes from young mice or 15 days to model adipocytes from old mice as described in previous studies [54, 80]. We confirmed that cells differentiated for 15 days expressed higher level of senescence marker p21 compared to those differentiated for 7 days. (Figure 4A). The aged 3T3-L1 adipocytes (more than 15 days differentiated cells) were treated with control ASO (100 μM) or CD47 ASO (100 μM) for 48 hrs to determine gene expression and *de novo* lipogenesis (DNL) assay.

In addition to determining the effect of CD47 ASO treatment on mature adipocyte function, as described above, we also investigated the effect of CD47 ASO on adipogenesis. Confluent 3T3-L1 cells were treated with either Control ASO (100 μM) or CD47 ASO (100 μM) in differentiation-inducing medium (DMEM containing 10% FBS, 25 mM HEPES, 4.5 g/L glucose, 100 U/mL penicillin/streptomycin, 0.5 mM IBMX, 1.7 μM insulin, and 1 μM dexamethasone) for 5 days, and the medium was replaced every other day. After 5 days of differentiation, cells were stained with Oil Red O (ORO) to assess lipid droplet formation, and the extracted ORO was quantified using a spectrophotometer at 492 nm. Total RNA from another set of cell plates was isolated and analyzed for gene expression by qPCR.

### *In vitro* DNL analysis

To perform DNL analysis, following control or CD47 ASO treatment, the aged 3T3-L1 adipocytes were pre-treated with lipolysis inhibitor, CAY10499 (10 μM) (Sigma Aldrich, St. Louis, MO, USA), for 1 hour. Then the cells were incubated for a further 4 hours with 30 mM of glucose. At the end of experiments, the cells were lysate by sonication in PBS and conditional medium were collected to analyze DNL and released FFA.

### Real-time quantitative PCR

Total RNA from the visceral white adipose tissue and other tissues was extracted, reverse transcribed to cDNA, subjected to quantitative PCR analysis using a MyiQ Real-time PCR Thermal Cycler (Bio-Rad, Hercules, CA, USA) with SYBR Green PCR Master Kit (Qiagen, Valencia, CA, USA) and normalized to β-actin mRNA levels as previously described [81]. All the primer sequences utilized in this study are listed in Table 1.

**Table 1:** Primer sequences for QPCR.

**Gene**	**Primer sequence**	**Genes**	**Primer sequence**
**Mouse primers**			
FASN	5′-TCCTGGAACGAGAACACGATCT-3′ 5′-GAGACGTGTCACTCCTGGACTTG-3′	SCD-1	5′-TTCTTGCGATACACTCTGGTGC-3′ 5′-CGGGATTGAATGTTCTTGTCGT-3′
ACC	5′-CCCAGCAGAATAAAGCTACTTTGG-3′ 5′-TCCTTTTGTGCAACTAGGAACGT-3′	SREBP1c	5′-GGAGCCATGGATTGCACATT-3′ 5′-ACAAGGGTGCAGGTGTCACC-3′
F4/80	5′-CTTTGGCTATGGGCTTCCAGTC-3′ 5′-GCAAGGAGGACAGAGTTTATCGTG-3′	IL-10	5′-GCTCTTACTGACTGGCATGAG-3′ 5′-CGCAGCTCTAGGAGCATGTG-3′
Nrf2	5′-CCAGCTACTCCCAGGTTGC-3′ 5′-CCAAACTTGCTCCATGTCCT-3′	MCP-1	5′-CAGCCAGATGCAGTTAACGC-3′ 5′-GCCTACTCATTGGGATCATCTTG-3′
IL-1β	5′-TGGAGAGTGTGGATCCCAAGCAAT-3′ 5′-TGTCCTGACCACTGTTGTTTCCCA-3′	TNFα	5′-AGCCGATGGGTTGTACCT-3′ 5′-TGAGTTGGTCCCCCTTCT-3′
TGFβ	5′-ACAATTCCTGGCGTTACC-3′ 5′-GGCTGATCCCGTTGATTT-3′	HSL	5′-GGCTCACAGTTACCATCTCACC-3′ 5′-GAGTACCTTGCTGTCCTGTCC-3′
AdipoQ	5′-AACATTCCGGGACTCTACT-3′ 5′-TACTGGTCGTAGGTGAAGAG-3′	CD36	5′-TTGTACCTGGGAGTTGGCGAGAAA-3′ 5′-ACAGTTCCGATCACAGCCCATTCT-3′
Glut1	5′-GCTGTGCTTATGGGCTTCTC-3′ 5′-AGAGGCCACAAGTCTGCAT-3′	ChREBP	5'-GGACAAGATCCGGCTGAACA-3' 5'-CGTCCGTTGCACATATTGAATG-3'
Glut2	5′-TGAGCAGAAGGTCTCCGTGA-3′ 5′-TGTCGGTAATTGGCATCCGT-3′	Acot1	5′-GACAAGAAGAGCTTCATTCCCGTG-3′ 5′-CATCAGCATAGAACTCGCTCTTCC-3′
Glut4	5′-CATGGCTGTCGCTGGTTTC-3′ 5′-AAACCCATGCCGACAATGA-3′	ATGL	5′-AACACCAGCATCCAGTTCAA-3′ 5′-GGTTCAGTAGGCCATTCCTC-3′
AP2	5′-TCACCTGGAAGACAGCTCCT-3′ 5′-AAGCCCACTCCCACTTCTTT-3′	CD47	5′-AGAATGCTTCTGGACTTGGCCTCA-3′ 5′-TCACATGCCATGATGCAGAGACAC-3′
LDLR	5′-GCTCCATAGGCTATCTGCTCTTCA-3′ 5′-GCGGTCCAGGGTCATCTTC-3′	Nrg4	5′-ATGCCAACAGATCACGAGC-3′ 5′-TCTTCAGTGTTCTCTGTGGCTG-3′
IRS	5′-CCTCAGTCCCAACCATAACCA-3′ 5′-CCGGCACCCTTGAGTGTCT-3′	UCP-1	5′-ACTGCCACACCTCCAGTCATT-3′ 5′-CTTTGCCTCACTCAGGATTGG-3′
FGF21	5′-GCTGCTGGAGGACGGTTACA-3′ 5′-CACAGGTCCCCAGGATGTTG-3′	CPT1α	5′-CTCTATGTGGTGTCCAAG-3′ 5′-CACAGGACACATAGTCAG-3′
G6PD	5′-CCTACCATCTGGTGGCTGTT-3′ 5′-CATTCATGTGGCTGTTGAGG-3′	P21	5′-CTGTCTTGCACTCTGGTGTCTGA-3′ 5′-CCAATCTGCGCTTGGAGTGA-3′
G6PC	5′-CCCAGGTTGAGTTGATCTTC-3′ 5′-GACTTCTTGTGTGTCTGTCC-3′	P27	5′-TCAGCGCAAGTGGAATTT-3′ 5′-GGGCCTGTAGTAGAACTCG-3′
PEPCK	5′-ACACCATCTTCACCAACG-3′ 5′-GTCTCCACTCCTTGTTCTTC-3′	P53	5′-AGTCCTTTGCCCTGAACTGC-3′ 5′-TTTACGCCCGCGGATCTTGA-3′
Arg1	5′-CTCCAAGCCAAAGTCCTTAGAG-3′ 5′-AGGAGCTGTCATTAGGGACATC-3′	Cidea	5′-AGGGACAACACGCATTTC-3′ 5′-GTAGGACACCGAGTACATCT-3′
C/EBPα	5′-CGCAAGAGCCGAGATAAAGC-3′ 5′-CGGTCATTGTCACTGGTCAACT-3′	Cox7α	5′-CAGCGTCATGGTCAGTCTGT-3′ 5′-AGAAAACCGTGTGGCAGAGA-3′
C/EBPβ	5′-TGATGCAATCCGGATCAAACGTGG-3′ 5′-TTTAAGTGATTACTCAGGGCCCGGCT-3′	CPT1β	5′-ACCTGAGCTGTGCTGAATAAA-3′ 5′-ACAGGAGACGGACACAGATA-3′
PPARγ	5′-TGCTGTTATGGGTGAAACTCTG-3′ 5′-CTGTGTCAACCATGGTAATTTCTT-3′	PPARα	5′-CAGCCTCAGCCAAGTTGAAG-3′ 5′-CGAACTTGACCAGCCACAAA-3′
β-actin	5′-GGCTGTATTCCCCTCCATCG-3′ 5′-CCAGTTGGTAACAATGCCATGT-3′	PGC1α	5′-CTGCATGAGTGTGTGCTGTG-3′ 5'-CAAATATGTTCGCAGGCTCA-3′

### Western blotting

Proteins obtained from WAT or liver were separated using sodium dodecyl sulfate (SDS)-polyacrylamide gel electrophoresis and subsequently transferred onto a nitrocellulose membrane. To determine protein expression, immunoblotting was performed using the anti-FASN antibody (Novus Biologicals), anti-p-AKT1 (Ser473, Cell Signaling Technology), anti-AKT1 (Cell Signaling Technology), anti-PKG-1 (Abcam), and anti-β-actin antibody (Sigma-Aldrich). The membranes were incubated with primary antibodies and then with appropriate secondary antibodies conjugated with horseradish peroxidase. Labelled proteins were visualized using an enhanced chemiluminescence system (Pierce, Waltham, MA, USA).

### Statistical analysis

Statistical analysis was performed using Prism version 9.0 (GraphPad Software, San Diego, CA, USA). Data are expressed as mean values ± SE. Student’s *t*-test was used to determine statistical significance between the two groups. One-way ANOVA followed by Bonferroni’s multiple comparisons test or two-way ANOVA followed by Tukey’s multiple comparisons test was used for multi-group comparisons.

## Supplementary Materials

Supplementary Figures
