# Supplementary material for: CD47 antisense oligonucleotide treatment improves glucose homeostasis and alleviates dyslipidemia in aged male mice
Source: Aging (Albany NY). 2025 Dec 1;17(12):2884–901. doi: 10.18632/aging.206343 (PMC13147726; doi:10.18632/aging.206343)
Supplement: Supplementary Figures [file aging-17-12-206343-s001.pdf]

SUPPLEMENTARY FIGURES

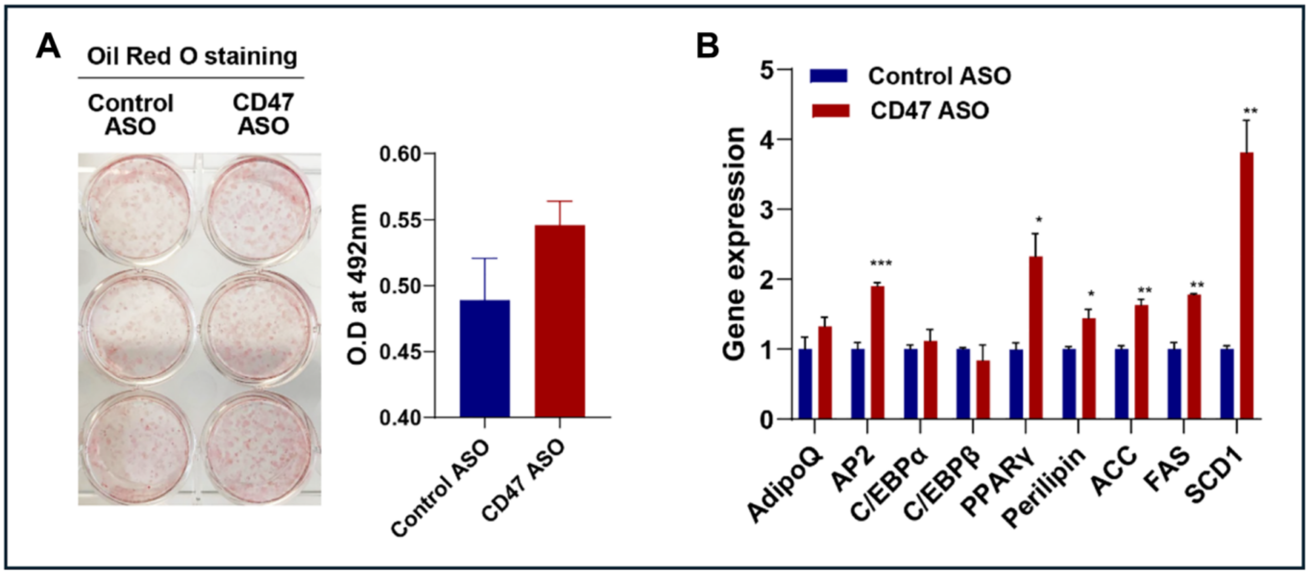

Supplementary Figure 1. Effect of CD47 ASO treatment on 3T3-L1 cell adipogenesis *in vitro*. (**A**) Confluent 3T3-L1 cells were treated with Control ASO or CD47 ASO (100 μM) in differentiation medium for 5 days, with medium replaced every other day. At the end of experiment, cells were stained with Oil Red O (ORO) to assess lipid droplet formation, and the extracted ORO was quantified using a spectrophotometer at 492 nm (**A**). Total RNA from another set of cell plates was isolated and analyzed for gene expression by qPCR (**B**). Data are represented as mean ± SEM (n = 3). $^{*}P < 0.05$, $^{**}P < 0.01$, and $^{***}P < 0.001$ compared to control ASO.

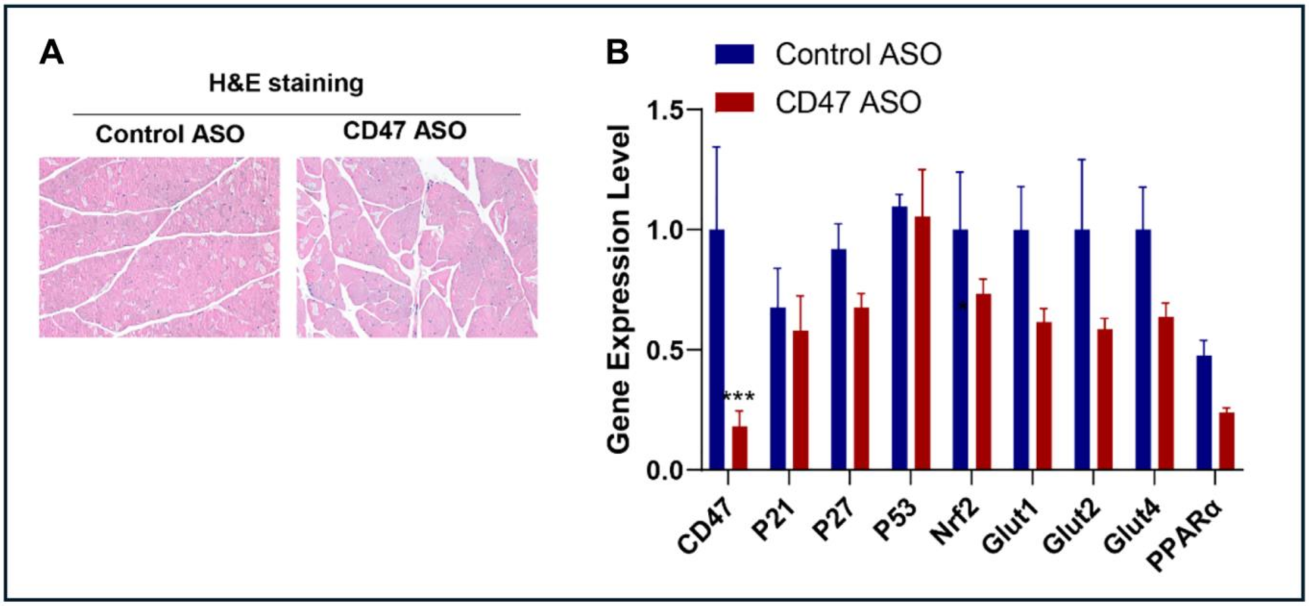

Supplementary Figure 2. Effect of CD47 ASO treatment on skeletal muscle histology and gene expression in aged male mice. (**A**) Representative H&E staining image of gastrocnemius muscle (scale bar = 100 μm); (**B**) Gene expression in gastrocnemius muscle by qPCR. Data are represented as mean ± SEM (n = 6 mice/group). $^{***}P < 0.001$ compared to control group.
